# Sporadic form of epidermolysis bullosa simplex with mottled pigmentation^☆^^☆☆^

**DOI:** 10.1016/j.abd.2019.08.033

**Published:** 2020-05-14

**Authors:** Flávia Regina Ferreira, Carolina Fernandes Pereira, Juliana Carvalho Moretto, Mariana Patriota Naville

**Affiliations:** Dermatology Service, Hospital Universitário de Taubaté, Taubaté, SP, Brazil

Dear Editor,

Epidermolysis bullosa (EB) composes a group of hereditary bullous disorders in which the blisters arise spontaneously or are triggered by minimal trauma. Koebner suggested this denomination in 1886. EB is divided into four major types (simplex, junctional, dystrophic, and Kindler syndrome) and several distinct clinical phenotypes, according to the level of skin cleavage, as well as clinical and molecular characteristics.^1^, ^2^

Epidermolysis bullosa simplex with mottled pigmentation (EBS-MP) is an uncommon subtype of epidermolysis bullosa simplex (EBS; Online Mendelian Inheritance in Man [OMIM] No. 131960). It is characterized by non-cicatricial blisters, mainly at the distal extremities, and progressive mottled hyperpigmentation. Until 2013, only 15 families and eight sporadic cases had been reported, according to the data from the Hospital Infantil Universitário Niño Jesus, in Madrid, which motivated this report.^2^

The patient was a 2-year-old girl, phototype III, with a history of blistering skin since birth. On dermatological examination she had desiccated blisters on the feet, as well as hyper- and hypochromic macules scattered over the tegument with mottled appearance (Figure 1, Figure 2). Normochromic papules on the dorsal region of the fingers and onychodystrophy were also seen. The blisters appeared spontaneously or after minimal trauma, according to the mother's report, and were located mainly at the distal extremities of the limbs. At two months of age, the hyper- and hypochromic macules began. The mother also referred episodes of oral mucositis. Immunomapping result (Fig. 3) coupled with the patient's clinical and laboratory findings confirmed the diagnosis of EBS-MP. This is probably a sporadic case since family history fo EB or other bullous disese is negative. The patient is under outpatient clinic follow-up. Family orientations were conducted in order to reduce the occurrence of new blisters and improve the coexistence of the patient with her genodermatosis.

**Figure 1:**
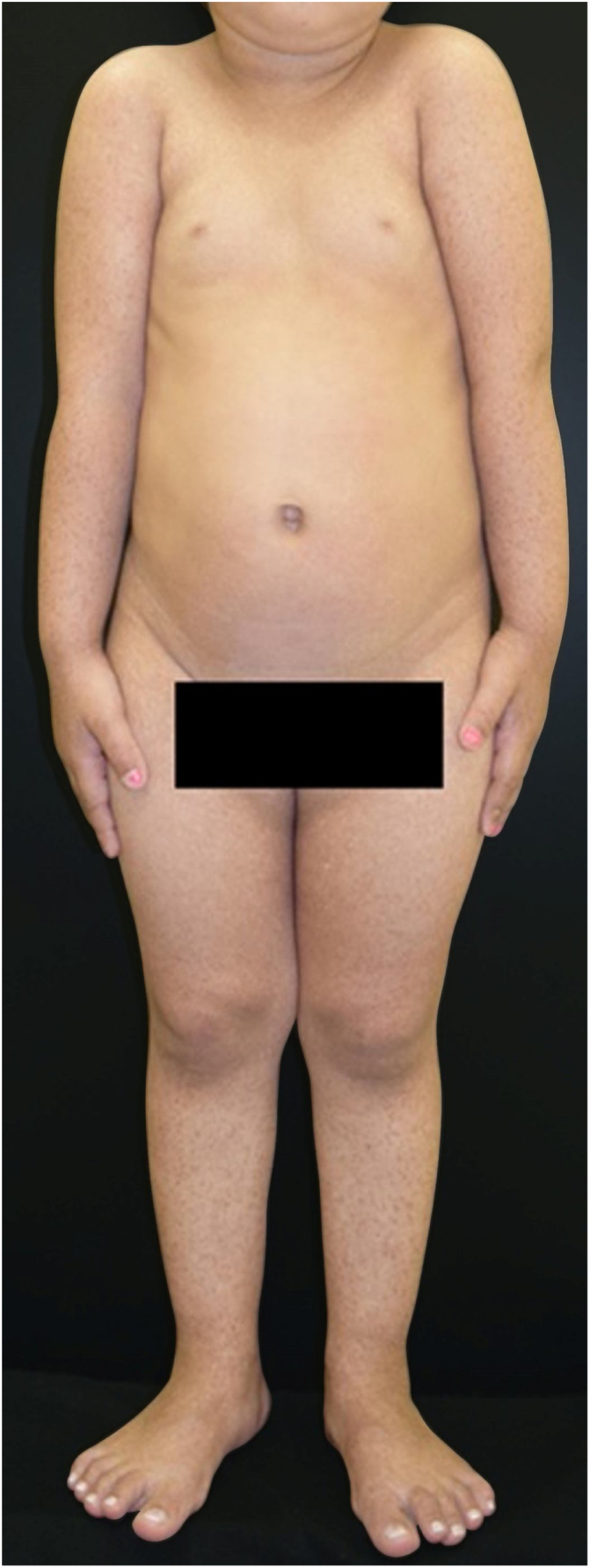
Disseminated hyper- and hypochromic macules scattered over the tegument with mottled appearance.

**Figure 2:**
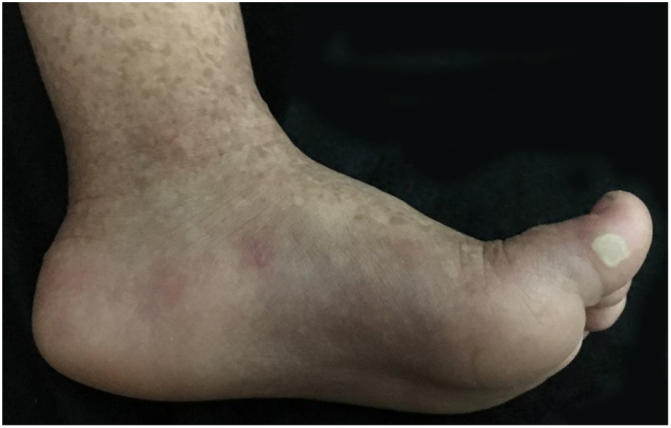
Close-up. Desiccated blisters and vesicles on the left foot.

**Figure 3:**
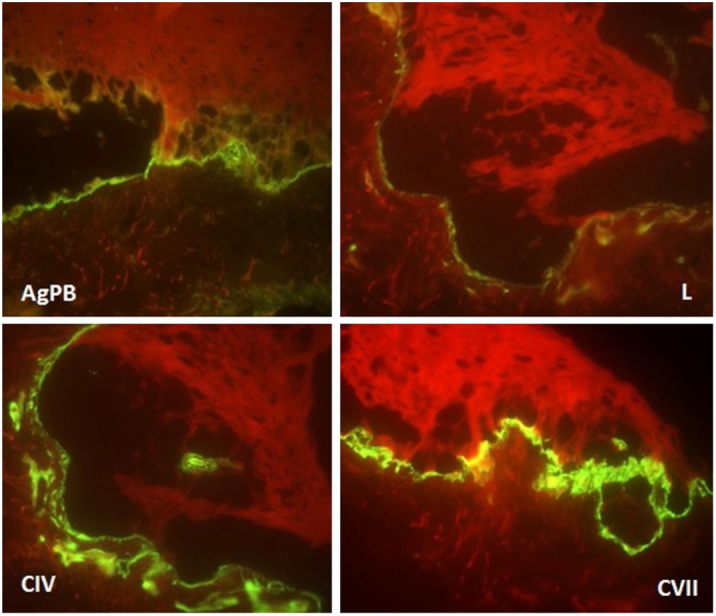
Immunomapping: fluorescence deposition on the blister floor (dermal side) observed with all antigenic markers (bullous pemphigoid antigen, laminin, collagens IV and VII).

First described in 1979 by Fischer and Gedd-Dahl, EBS-MP begins in childhood and has a genetic origin. It is a basal EBS caused by a mutation in the KRT5 gene that encodes cytokeratin 5. It occurs most commonly due to a punctual heterozygous p24L mutation in the non-helical V1 domain of cytokeratin 5.^1^, ^2^

The diagnosis of this dermatosis is based on typical clinical findings, family history, immunomapping, and/or transmission electron microscopy, as well as molecular/mutation analysis when possible.^2^

Clinically, it is characterized by non-cicatricial blisters, mainly at the distal extremities, as well as progressive mottled hyperpigmentation, which does not occur at the site of the blisters and often disappears in adulthood. Some cases may be accompanied by hypopigmented macules, as could be seen in this patient. There are also reports of palmar and plantar focal hyperkeratosis. Small acral verrucous papules, onychodystrophy, and mild involvement of the oral mucosa can be observed during childhood. Uncommon findings include photosensitivity and dental disorders (caries).^2^

The differential diagnosis of EBS-MP includes other types of EBS (mainly the herpetiformis type of Dowling-Meara), Kindler syndrome, Naegeli-Franceschetti-Jadassohn (NFJ) ectodermal dysplasia, other forms of dyschromia, Dowling-Degos disease, and even atypical cases of Darier's disease with mutations in ATP2A2.^2^, ^3^, ^4^

Due to the clinical hypothesis of EB and to determine the level of skin cleavage, immunomapping or transmission electron microscopy should be performed. The immunomapping has diagnostic accuracy similar to transmission electron microscopy, with the advantage of simple and fast execution and reading. It is associated with the use of monoclonal antibodies and may be considered an indirect immunofluorescence technique. In the EBS, the skin cleavage occurs in the basal layer (intra-epidermal), and fluorescence deposition on the blister floor (dermal side) is seen with all antigenic markers (bullous pemphigoid antigen, laminin, collagens IV and VII), as observed in this case.^5^

Ultrastructural analysis of the pigmented areas in this form of EBS demonstrates abundant mature melanosomes within the basal cells.^2^

Thus, this report details a rare case of a possibly sporadic EBS-MP. The authors emphasize the rarity of this subtype of EBS and its remarkable clinical characteristics favoring future diagnoses, and highlight its benign character, with no scarring or deforming lesions and regression of hyperpigmentation in adulthood.

## Financial support

None declared.

## Authors’ contributions

Flávia Regina Ferreira: Approval of final version of the manuscript; conception and planning of the study; drafting and editing of the manuscript; collection, analysis, and interpretation of data; intellectual participation in the propaedeutic and/or therapeutic conduct of the studied cases; critical review of the literature; critical review of the manuscript.

Carolina Fernandes Pereira: Approval of final version of the manuscript; conception and planning of the study; drafting and editing of the manuscript; critical review of the literature.

Juliana Carvalho Moretto: Approval of final version of the manuscript; conception and planning of the study; drafting and editing of the manuscript; critical review of the literature.

Mariana Patriota Naville: Approval of final version of the manuscript; conception and planning of the study; drafting and editing of the manuscript; critical review of the literature.

## Conflicts of interest

None declared.
